# Genomes of Geoalkalibacter ferrihydriticus Z-0531^T^ and Geoalkalibacter subterraneus Red1^T^, Two Haloalkaliphilic Metal-Reducing *Deltaproteobacteria*

**DOI:** 10.1128/genomeA.00039-15

**Published:** 2015-03-12

**Authors:** Jonathan P. Badalamenti, Rosa Krajmalnik-Brown, César I. Torres, Daniel R. Bond

**Affiliations:** a BioTechnology Institute, University of Minnesota, Saint Paul, Minnesota, USA; b Swette Center for Environmental Biotechnology, Biodesign Institute, Arizona State University, Tempe, Arizona, USA; c Department of Microbiology, University of Minnesota, Saint Paul, Minnesota, USA

## Abstract

We sequenced and annotated genomes of two haloalkaliphilic *Deltaproteobacteria*, Geoalkalibacter ferrihydriticus Z-0531^T^ (DSM 17813) and Geoalkalibacter subterraneus Red1^T^ (DSM 23483). During assembly, we discovered that the DSMZ stock culture of G. subterraneus was contaminated. We reisolated G. subterraneus in axenic culture and redeposited it in DSMZ and JCM.

## GENOME ANNOUNCEMENT

While complete genomes exist for several freshwater sediment and subsurface isolates from the *Geobacteraceae* and *Desulfuromonadaceae* families of *Deltaproteobacteria* (1), genomes from haloalkaliphilic members capable of metal reduction and electrode respiration at elevated salinity or pH (2–5) are lacking. To facilitate genetic studies of extracellular electron transfer in these haloalkaliphiles, we assembled and annotated draft and finished genomes of Geoalkalibacter ferrihydriticus Z-0531 and Geoalkalibacter subterraneus Red1, respectively.

For G. ferrihydriticus, we used the a5 pipeline (26 March 2013 release) (6) to assemble a 3,839,416-bp draft genome in 23 contigs at ~100× coverage (*N*_50_ = 602,663 bp; 57.95% GC) using 2 × 100 bp paired-end Illumina reads. More than 99% of the assembled sequence exists in 14 contigs, with the largest contig (1,394,064 bp) comprising >36% of the assembly.

Applying the same approach to G. subterraneus, multiple occurrences of single-copy genes and contigs with low G+C content indicated that the stock culture (5) was contaminated with a Gram-positive organism related to *Tieserella* sp. This finding was subsequently confirmed by DSMZ (S. Spring, personal communication). We reisolated G. subterraneus on solid medium lacking yeast extract and performed PacBio long-read sequencing according to protocols for a 20-kb insert size. SMRT bell templates were size-selected with a 4-kb cutoff using Blue Pippin electrophoresis (Sage Science). Subread filtering from 4 SMRT cells (P4-C2 chemistry, 120-min movies) yielded 1.13 Gbp of sequence (average read length = 4,760 bp; *N*_50_ = 6,620 bp). Assembly was performed using HGAP version 2 (7) with default parameters in SMRT Analysis version 2.1. One 400-Mbp SMRT cell was sufficient to assemble the G. subterraneus chromosome into one linear contig, but long reads from four SMRT cells were required to resolve three tandem copies of the rRNA operon (total length 16,597 bp) and circularize the contig. The assembly was polished to >99.999% consensus concordance (QV 50) with three successive passes through Quiver (7) at 250× coverage, and remaining indels were removed with Pilon version 1.8 (8) using 100× coverage of 2 × 100-bp paired-end Illumina reads. The finished assembly consisted of one chromosome (3,475,523 bp) and one megaplasmid (242,122 bp) totaling 3,717,645 bp (G+C content 56.68%).

Annotation via the NCBI Prokaryotic Genome Annotation Pipeline revealed features consistent with other *Geobacteraceae* and *Desulfuromoadaceae* genomes, including a complete tricarboxylic acid cycle with a eukaryotic-like citrate synthase (9) and an abundance of putative histidine kinases (>40 in both genomes) and multiheme *c*-type cytochromes for extracellular respiration (43 and 51 in G. ferrihydriticus and G. subterraneus, respectively) (1). Notably, only G. ferrihydriticus contains a putative *hgcAB* gene cluster indicative of mercury methylation (GFER_06575 and GFER_06580) (10).

### Nucleotide sequence accession numbers.

G. subterraneus has been redeposited in DSMZ and JCM. Sequences have been deposited in GenBank under the accession numbers JWJD00000000 (G. ferrihydriticus) and CP010311 and CP010312 (G. subterraneus chromosome and plasmid, respectively). Raw Illumina and PacBio reads, as well as base modification data for G. subterraneus, have been deposited to the NCBI Sequence Read Archive under accession numbers SRX808753 and SRX808316 for G. ferrihydriticus and G. subterraneus, respectively.
